# Efficient and Moisture‐Stable Inverted Perovskite Solar Cells via n‐Type Small‐Molecule‐Assisted Surface Treatment

**DOI:** 10.1002/advs.202205127

**Published:** 2022-11-23

**Authors:** Ji A Hong, Mingyu Jeong, Sujung Park, Ah‐Young Lee, Hye Seung Kim, Seonghun Jeong, Dae Woo Kim, Shinuk Cho, Changduk Yang, Myoung Hoon Song

**Affiliations:** ^1^ Department of Materials Science and Engineering Ulsan National Institute of Science and Technology (UNIST) Ulsan 44919 Republic of Korea; ^2^ Department of Energy Engineering School of Energy and Chemical Engineering Perovtronics Research Center Low dimensional Carbon Materials Center Ulsan National Institute of Science and Technology (UNIST) Ulsan 44919 Republic of Korea; ^3^ KEPCO Research Institute Korea Electric Power Corporation 105, Munji‐ro, Yuseong‐gu Daejeon 34056 Republic of Korea; ^4^ Department of Physics and EHSRC University of Ulsan Ulsan 44610 Republic of Korea; ^5^ Graduate School of Carbon Neutrality Ulsan National Institute of Science and Technology (UNIST) 50 UNIST‐gil, Ulju‐gun Ulsan 44919 Republic of Korea

**Keywords:** additive engineering, charge extraction, defect passivation, moisture stability, perovskite solar cells

## Abstract

Defect states at the surface and grain boundaries of perovskite films have been known to be major determinants impairing the optoelectrical properties of perovskite films and the stability of perovskite solar cells (PeSCs). Herein, an n‐type conjugated small‐molecule additive based on fused‐unit dithienothiophen[3,2‐*b*]‐pyrrolobenzothiadiazole‐core (JY16) is developed for efficient and stable PeSCs, where JY16 possesses the same backbone as the widely used Y6 but with long‐linear *n*‐hexadecyl side chains rather than branched side chains. Upon introducing JY16 into the perovskite films, the electron‐donating functional groups of JY16 passivate defect states in perovskite films and increase the grain size of perovskite films through Lewis acid–base interactions. Compared to Y6, JY16 exhibits superior charge mobility owing to its molecular packing ability and prevents decomposition of perovskite films under moisture conditions owing to their hydrophobic characteristics, improving the charge extraction ability and moisture stability of PeSCs. Consequently, the PeSC with JY16 shows a high power conversion efficiency of 21.35%, which is higher than those of the PeSC with Y6 (20.12%) and without any additive (18.12%), and outstanding moisture stability under 25% relative humidity, without encapsulation. The proposed organic semiconducting additive will prove to be crucial for achieving highly efficient and moisture stable PeSCs.

## Graphical abstract

A newly designed fused‐unit dithienothiophen[3,2‐*b*]‐pyrrolobenzothiadiazole‐core‐based n‐type conjugated small molecule, JY16, is developed for application in perovskite solar cells (PeSCs) via anti‐solvent dropping method. JY16 suggests its potential as an ideal additive for highly efficient and moisture‐stable PeSCs owing to its excellent mobility, defect passivation, and moisture‐repellent abilities.

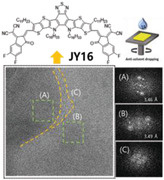

## 1 Introduction

The adoption of metal halide‐based perovskite solar cells (PeSCs) has been rapidly increasing owing to their outstanding photoelectric properties such as longer carrier diffusion,^[^ ^1^ ^]^ high optical absorption coefficient,^[^ ^2^ ^]^ and low fabrication costs. In particular, inverted‐structured (p‐i‐n) PeSCs possess reproducibility with negligible hysteresis, making them promising candidates for commercial adoption. Because of their polycrystalline nature, solution‐processed perovskite films contain inevitable defects such as vacancies, interstitials, and antisites of perovskite films at their surface and/or grain boundaries.^[^ ^3^ ^]^ These defects including uncoordinated Pb^2+^ accelerate the decomposition of perovskite films, causing non‐radiative charge recombination, which ultimately deteriorates the performance and stability of PeSCs.^[^ ^4^ ^]^ With continuous improvements in power conversion efficiency (PCE) of PeSCs in recent years, considerable efforts have been devoted to passivating the grain boundaries of perovskite films with large gain sizes using passivating additives, and further improving the device stability and efficiency.

In addition, the crystal growth kinetics of perovskite can be properly modulated by additives such as polymers, ammonium salts, low‐dimensional perovskites, Lewis acids, Lewis bases, and ionic liquids.^[^ ^3^, ^5^ ^]^ The growth of perovskite can be regulated by incorporating Lewis base semiconducting materials with electron‐donating functional groups such as sulfur (S), nitrogen (N), and oxygen (O) into the perovskite films to take advantage of Lewis acid–base interactions between the additive material and perovskite layer, resulting in large grain‐size perovskite films with high crystallinity.^[^ ^5^ ^]^ Moreover, the insertion of Lewis base semiconducting additives with electron‐donating functional groups into perovskite can passivate the defect states of perovskite films, thereby further enhancing the PCE and stability of PeSCs.^[^ ^6^ ^]^ For example, Yu et al. used an organic semiconducting additive, SA‐2, in the perovskite film to control the grain size and reduce its defect states, resulting in 20.3% of PCE and maintained 84% of its initial efficiency under continuous illumination (1sun, 1000 mW cm^−2^) at atmospheric condition (25 °C, 60% RH) over 170 h.^[^ ^7^ ^]^ Koo et al. used an organic semiconducting additive, Y‐Th2, in the perovskite film to minimize the defect states and improve the operational stability of PeSCs, resulting in 21.5% of PCE and maintained 82.8% of its initial efficiency under ambient air condition (25 °C, 40% RH) over 1600 h.^[^ ^8^ ^]^ In addition, Chen et al. introduced the polymeric semiconducting additive PBTI to passivate the grain boundaries of perovskite in inverted‐structured PeSCs, resulting in 20.67% of PCE and maintained 70% of its initial PCE under 1sun condition in nitrogen environment over 600 h.^[^ ^9^ ^]^

Evidently, semiconducting additives containing Lewis base functional groups efficiently contribute to mitigating the defect states of perovskite films. However, when considering the significance of commercial viability, it is important to choose additives that can maximize charge extraction and stability while maintaining defect passivation capability.

Many recent studies have reported that side chain engineering of semiconducting materials has been successfully employed to improve optoelectronic device performance, advocating its important roles beyond just solubilizing groups.^[^ ^10^ ^]^ In this regard, it is well known that replacing the branched side chains with linear ones can manipulate the molecular packing ability, leading to enhanced electrical properties.^[^ ^11^ ^]^ In addition, because of the nature of perovskite films, which are vulnerable to moisture, suppressing the infiltration of moisture from the external environment is a crucial factor in delaying the degradation of perovskite films.^[^ ^12^ ^]^ In this regard, adjusting the length of the side chain is suitable for increasing hydrophobicity, leading to enhanced moisture stability or PeSCs.^[^ ^13^ ^]^

In this study, we synthesized an n‐type conjugated small‐molecule additive based on the fused‐unit dithienothiophen[3,2‐*b*]‐pyrrolobenzothiadiazole‐core (JY16) for efficient and stable PeSCs. JY16 has the same backbone with electron‐donating group as Y6, which is widely used as an additive and interfacial layer in PeSCs.^[^ ^14^ ^]^ JY16 is effective for controlling the defect states of perovskite films through Lewis interactions between the Lewis acid of perovskite and Lewis base functional groups in JY16. In addition, the long‐linear *n*‐hexadecyl side chains of JY16 can manipulate the molecular packing ability, leading to enhanced electrical properties and increased hydrophobicity, which further leads to enhanced moisture stability. JY16 and Y6 were introduced into perovskite films as organic semiconducting additives via the anti‐solvent dropping method, and inverted‐structured PeSCs with JY16 and Y6 additives were fabricated and compared. The PeSCs with JY16 additive showed better charge transport and extraction ability because of the superior mobility of JY16 originating from the strong interchain interdigitation and p‐interactions induced by all linear side chains. As a result, we achieved best PCE of 21.35% with insignificant hysteresis from PeSCs with JY16 additive. The PeSCs with JY16 additive also exhibited excellent device stability, showing 76% PCE retention after 500 h under 25% RH without any encapsulation.

## 2 Results and Discussion

**Figure 1:**
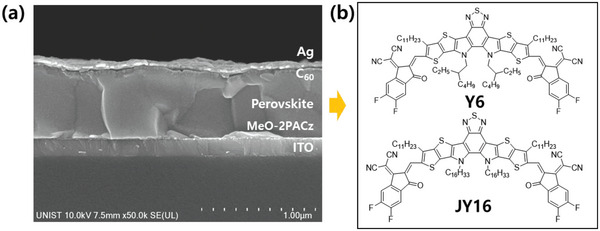
a) Cross‐sectional SEM images of PeSC with JY16 additive. b) Chemical structure of Y6 and JY16.

Cross‐sectional scanning electron microscopy (SEM) images of the inverted‐structured PeSCs with JY16 and Y6 treatment and without treatment (control) were measured to examine the cross‐sectional morphology of the devices (**Figure** **1**a and Figure S1, Supporting Information). The device is composed of a glass substrate, ITO as anode, self‐assembled monolayer MeO‐2PACz as hole transport layer, Cs_0.05_(FA_0.92_MA_0.08_)_0.95_Pb(I_0.92_Br_0.08_)_3_ as perovskite film, LiF/C_60_ as electron transport layer (ETL), and Ag as cathode. Similar to Y6, JY16 has sufficient solubility in organic solvents used as anti‐solvents of dropping method such as chlorobenzene, toluene, and chloroform (Figure S2, Supporting Information). A 450 nm‐thick perovskite film was fabricated via an anti‐solvent (chloroform, in our study) dropping method, and a small amount of Y6 or JY16 present in the anti‐solvent was dropped on the perovskite precursor while the spin‐coating procedure. The details of the fabrication procedure are described in the Experimental Section in Supporting Information. The molecular structures and synthetic procedures are shown in Figure 1b and Figure S3, Supporting Information. Y6 and JY16, which contain electron‐donating functional groups such as S, N, and O can passivate the defect states of perovskite films such as Pb^2+^, and the formation of Lewis acid–base adducts can affect perovskite film growth.

**Figure 2:**
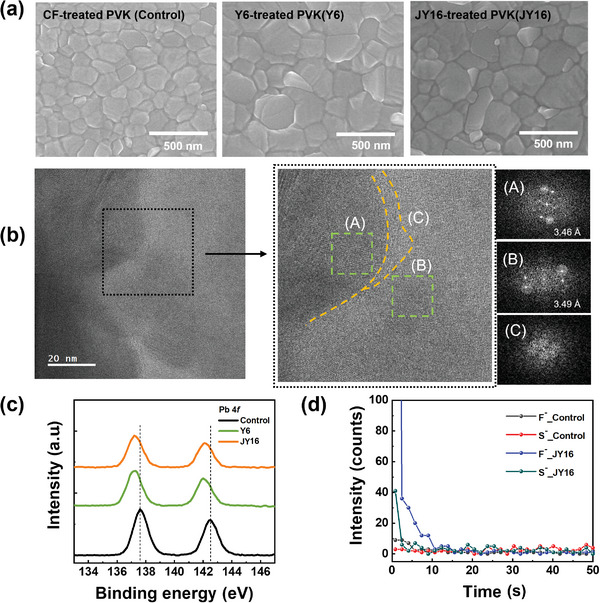
a) Top‐view SEM images of control, Y6‐, and JY16‐treated perovskite films. b) HRTEM of JY16‐treated perovskite film. c) XPS spectra of Pb 4f for control, Y6‐, and JY16‐treated perovskite films. d) TOF‐SIMS spectra of JY16‐treated perovskite film.

To demonstrate the effect of the molecular structure of the Lewis bases on the formation and growth of perovskite films, top‐view SEM images of the control, Y6‐, and JY16‐treated perovskite films were obtained (**Figure** **2**a). The grain size of the Y6‐ and JY16‐treated perovskite films increased via Lewis acid–base interactions between Pb^2+^ of perovskite and Lewis base functional groups in Y6 and JY16, which slowed nucleation and retarded the growth of perovskite films.^[^ ^15^ ^]^ SEM was used to investigate the morphology of the JY16‐treated perovskite films according to varied JY16 concentrations (Figure S4, Supporting Information). Perovskite films treated with JY16 of various concentrations exhibited morphology with densely packed and free of pinholes. It is noteworthy that there is an obvious grain size difference among all the films with various concentrations of JY16 owing to Lewis acid–base interactions. A higher amount of JY16 in perovskite leads to a larger grain size in perovskite films, reducing grain boundaries and their associated defects. The crystallinity and crystal orientation of the control, Y6‐, and JY16‐treated perovskite films were analyzed using X‐ray diffraction (XRD) (Figure S5, Supporting Information). No significant differences were observed in the crystal lattice or diffraction patterns of the control, Y6‐, and JY16‐treated perovskite films. However, the integrated intensity of the XRD peaks at 2*θ* = 12.6° assigned to PbI_2_, compared to the XRD peaks at 2*θ* = 14.1° assigned to the (110) plane of perovskite, was found to slightly decrease in the Y6‐ and JY16‐treated perovskite films compared to the control film, indicating that Y6 and JY16 affect the improvement in perovskite film quality. To further investigate the Lewis acid–base interactions between the perovskite film and the additive, X‐ray photoelectron spectroscopy (XPS) analysis was performed (Figure 2c and Figure S6, Supporting Information). The control perovskite film exhibited the binding energy of the Pb 4f_7/2_ at 137.5 and 142.4 eV, whereas both the Y6 and JY16‐treated perovskite films showed lower binding energy for Pb 4f_7/2_ at 137.3 and 142.2 eV. The shift in the binding energy to a lower value is due to the coordination bond between the Pb^2+^ of the perovskite precursor and the Lewis base functional units with a high electron density.^[^ ^16^ ^]^ To further determine which electron‐donating functional atoms in Y6 and JY16 interact with perovskite, XPS spectra of S, O, and N of control, Y6‐, and JY16‐ treated perovskite films, pristine Y6, and JY16 were measured (Figure S6, Supporting Information). The N peaks in C=NH_2_^+^ of perovskite and pyrrolic of Y6 and JY16 were overlapped around at 400 eV, and the cyano groups of Y6‐, and JY16‐treated perovskite films was clearly observed at 398.2 and 397.9 eV, shifting toward higher binding energies compared to pristine Y6 and JY16 (398.1 and 397.7 eV, respectively).^[^ ^14^ ^]^ Electron‐rich cyano groups of Y6 and JY16 contribute to donate electrons to Pb^2+^ of perovskite, resulting in a shift to higher binding energies. Pristine Y6 and JY16 exhibited the binding energies of carbonyl peaks of O at 530.6 and 530.5 eV, whereas Y6‐ and JY16‐treated perovskite films showed the binding energy of carbonyl peaks at 530.9 and 531.3 eV, shifting toward higher binding energies.^[^ ^14^ ^]^ Furthermore, the S 2p_3/2_ and 2p_1/2_ peaks of Y6‐ and JY16‐treated perovskite films shifted toward higher binding energies compared to pristine Y6 and JY16, revealing that electron rich Lewis base functional groups of carbonyl and C‐S‐C of Y6 and JY16 are properly involved in the interaction with Pb^2+^ of perovskite films.^[^ ^14^ ^]^

In addition, we investigated the distribution of JY16 in the perovskite films by measuring the time‐of‐flight secondary‐ion mass (TOF‐SIMS) depth profiling of the control and JY16‐treated perovskite films (Figure 2d). The negative ions S^−^ and F^−^ were used as indicators of JY16. The JY16‐treated perovskite film showed both S^−^ and F^−^ signals in the early seconds, whereas the control perovskite film did not show any S^−^ and F^−^ signals, indicating that JY16 is mostly distributed on the upper inner and/or upper surface of the perovskite film. High‐resolution transmission electron microscopy (HRTEM) was used to further validate the presence of JY16 in the perovskite film (Figure 2b), and three highlighted regions of the magnified image were analyzed: A,B) the inner region of the perovskite grains and C) the grain boundary region between regions (A) and (B). Fast Fourier transform (FFT) analysis inside the perovskite grains (regions [A] and [B]) revealed interplanar spacing of 3.46 and 3.49 Å, respectively, indicating the (002) reflection of the perovskite films.^[^ ^17^ ^]^ On the contrary, the FFT image of (C) located at the perovskite grain boundary showed amorphous diffraction patterns, inferring JY16. The results from TOF‐SIMS depth profiling and HRTEM measurements support that JY16 is located not only on the upper surface but also at the grain boundary of the upper inner perovskite films. Because uncoordinated Pb^2+^ defects tend to exist at the grain boundaries and/or both surfaces of perovskite films, JY16 and Y6 can be considered appropriate candidates for reducing the non‐radiative recombination process caused by defect states in perovskite films.

**Figure 3:**
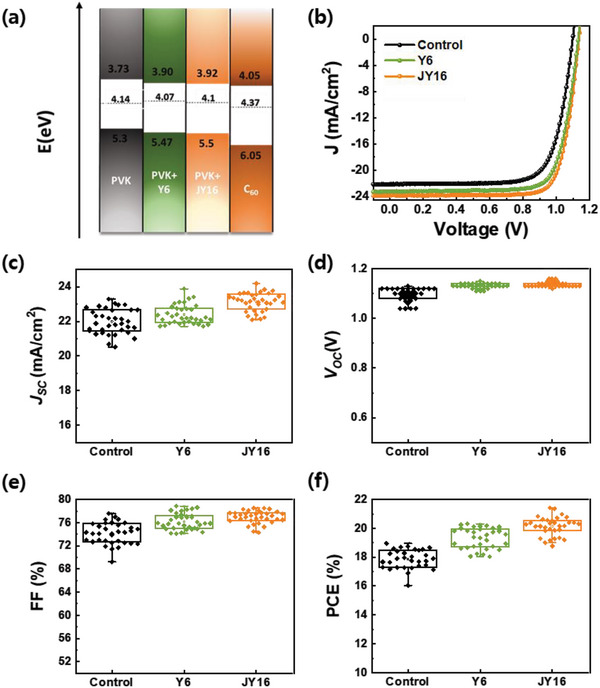
a) Energy level diagram of control, Y6‐, and JY16‐treated perovskite films and ETL. b) *J*–*V* characteristics of best‐performed control PeSC and PeSCs with Y6 and JY16 additives. Histogram of c) *J*_sc_, d) *V*_oc_, e) FF, and f) PCE from control PeSC and PeSCs with Y6 and JY16 additives.

To investigate the charge extraction ability of PeSCs, we obtained the energy levels of the control, Y6‐, and JY16‐treated perovskite films using ultraviolet photoelectron spectroscopy analysis (**Figure** **3**a and Figure S7, Supporting Information). The work function of the control, Y6‐, and JY16‐treated perovskite films were determined to be 4.14, 4.07, and 4.1 eV via the secondary cut‐off region, respectively. Considering the optical bandgap of the control, Y6‐, and JY16‐treated perovskite films (Figure S7c, Supporting Information), their LUMO levels were obtained to be 3.73, 3.90, and 3.92 eV, respectively. Electron extraction from Y6‐ and JY16‐treated perovskite films to the ETL (C_60_) appears to be preferable.

The best‐performed *J*–*V* characteristics of control PeSC and PeSCs with JY16 and Y6 additives of backward scanning directions under 25 °C with 100 mW cm^−2^ of incident light intensity (Figure 3b) are shown, and the hysteresis behavior of control and PeSCs with JY16 and Y6 additives (Figure S8, Supporting Information) are shown as well. To optimize the device performance of PeSCs with the JY16 additive, PeSCs with different amounts of JY16 in the perovskite were fabricated and compared (Figure S9 and Table S1, Supporting Information). The best PeSC with JY16 additive showed enhanced device performance, compared to control PeSC and PeSC with Y6 additive, showing a short‐circuit current density (*J*_SC_) of 23.84 mA cm^−2^, an open‐circuit voltage (*V*_OC_) of 1.14 V, a fill factor (FF) of 78.54%, and a PCE of 21.35% (**Table** **1**) and the corresponding stabilized power output is shown (Figure S10, Supporting Information). More than 30 identical devices were fabricated and measured in order to assess the repeatability of the control, Y6‐, and JY16‐treated PeSCs (Figure 3c–f). The best‐performed values, average values, and standard deviations of the photovoltaic factors, PCE, *J*_SC_ *, V*_OC_, and FF are exhibited in Table 1. The average photovoltaic values of the PeSCs treated with JY16 was improved than those of the control PeSC and PeSCs with Y6 additives, indicating superior reproducibility with a small standard deviation. First, to identify the higher *J*_SC_ in the PeSC with JY16, the incident photon‐to‐electron conversion efficiency (IPCE) spectra for the corresponding control PeSC and PeSCs with JY16 and Y6 additives were measured (Figure S11, Supporting Information). Photocurrent values of the control PeSC and PeSCs with JY16 and Y6 additives obtained by integrating the region below the IPCE spectra were 22.10, 22.93, and 23.13 mA cm^−2^, which are correspond to the *J*_SC_ values determined from the *J*–*V* characteristics. The primary reason why the PeSC with JY16 additive shows a higher *J*_SC_ is explained later. The FF and *V*_OC_ of the PeSCs with JY16 and Y6 additives were significantly enhanced compared to those of the control device, which was primarily caused by the reduction of defect states in the perovskite films and interfaces using Y6 and JY16 additives with Lewis base functional groups.

**Table 1:** Summary of best‐performed value with standard deviation and average value of key factors of the control PeSC and PeSCs with Y6 and JY16 additives

Device configuration	*J*_sc_ [mA cm^−2^]	*V*_oc_ [V]	FF [%]	PCE [%]
Control	22.12^a)^ (21.99±0.713)^b)^	1.1 (1.1±0.024)	74.49 (74.15±1.847)	18.12 (17.86±0.678)
Y6	23.24 (22.39±0.551)	1.14 (1.13±1)	76.28 (76.21±1.381)	20.12 (19.29±0.739)
JY16	23.85 (23.12±0.52)	1.14 (1.14±0.01)	78.54 (76.94±1.092)	21.35 (20.24±0.60)

^a)^ Best cell photovoltaic parameters of control PeSC and the PeSCs with Y6 and JY16 additives;

^b)^ Values in parentheses are average values and standard deviation from 30 cells of control PeSCs and the PeSCs with Y6 and JY16 additives.

**Figure 4:**
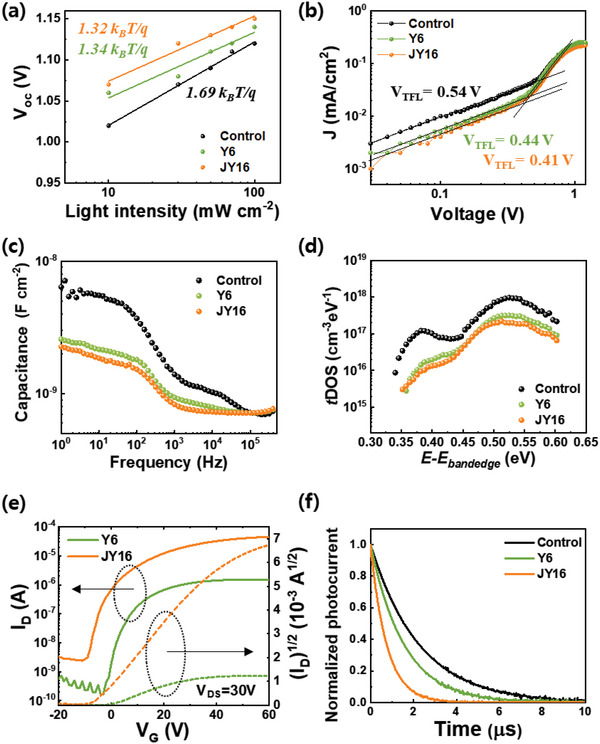
a) Light‐intensity dependent *V*_oc_ of control PeSC and PeSCs with Y6 and JY16 additives. b) Hole‐only device of control PeSC and PeSCs with Y6 and JY16 additives (glass/ITO/PTAA/CPE/perovskite/Au). c) Frequency‐dependent capacitance measurements and d) trap density of control PeSC and PeSCs with Y6 and JY16 additives from frequency‐dependent capacitance spectra. e) FETs with Y6 and JY16 additives. f) TPC of control PeSC and PeSCs with Y6 and JY16 additives.

To identify the lowered defect states with the addition of Y6 or JY16 in perovskite films, the dominant types of recombination processes in PeSCs were confirmed by measuring the ideality factor of the control, Y6‐, and JY16‐treated PeSCs. The variation in *V*_OC_ depending on light intensity was measured using Equation (1):

(1) $$V_{\mathrm{OC}}=\frac{nkTln\left(I\right)}{q}+c$$

where *n* is an ideal factor for inferring the dominant recombination type, *k* is the Boltzmann constant, *T* is the absolute temperature, *I* is the light intensity, *q* is the elementary charge, and *c* is the fitting constant, representing all values independent of the light intensity. The *n* values were reduced from 1.69 for the control PeSC to 1.34 and 1.32 for the Y6‐ and JY16‐treated PeSCs, respectively. The lower ideality factors of the PeSCs with Y6 and JY16 additives compared to the control PeSC exhibit that the trap‐assisted recombination was effectively reduced with Y6 and JY16 additives (**Figure** **4**a). Moreover, the defect passivation effects of Y6 and JY16 were demonstrated by comparing the trap‐filled‐limited‐voltage (*V*_TFL_) of hole‐only devices (glass/ITO/MeO‐2PACz/perovskite with additives/Au) for the control, Y6‐, and JY16 treatment (Figure 4b). Generally, the dark *J*–*V* curve is divided into Ohmic, trap‐filled limited, and space‐charge‐limited‐current regions. *V*_TFL_ is determined by the point between the Ohmic and trap‐filled limited regions. The *V*_TFL_ values of the control device and Y6‐ and JY16‐treated perovskite devices are 0.54, 0.44, and 0.41 V, respectively, indicating that the trap densities of perovskite films were effectively reduced by Y6 and JY16 treatment.

The reduction of defect states in the perovskite films by Y6 or JY16 treatment was further confirmed by capacitance‐frequency measurements (Figure 4c).^[^ ^5b^ ^]^ The distribution of the trap density of state (tDOS) was obtained from the derivative of the capacitance with respect to the frequency according to Equation (2):^[^ ^18^ ^]^

(2) $$N_{t}=-\frac{V_{\mathrm{bi}}}{qW}\frac{dC}{d\omega }\frac{\omega }{k_{B}T}$$

where *C* is the capacitance, *ω* is the angular frequency, *q* is the elementary charge, *k*_B_ is the Boltzmann's constant, *T* is the temperature, and *W* is the depletion width. The built‐in potential (*V*_bi_) was obtained using Mott–Schottky analysis (Figure S12, Supporting Information). The applied angular frequency *ω* defines the energetic demarcation, according to Equation (3):

(3) $$E_{\omega }=k_{B}T\;ln\left(\frac{\omega_{0}}{\omega }\right)$$

where *ω*_0_ is the attempt‐to‐escape frequency, which was assumed to be ≈10^11^ s^−1^.^[^ ^18^ ^]^ Because trap states below the energy demarcation can either trap or release charge with a given *ω* and contribute to the capacitance, the frequency differential capacitance provides the distribution of the tDOS of the perovskite films.^[^ ^19^ ^]^ Both tDOS of the shallow trap (0.35–0.45 eV) and deep trap (0.50–0.55 eV) were significantly reduced after treatment of perovskite films with Y6 and JY16, which presents that both Y6 and JY16 effectively passivated the defects in perovskite films (Figure 4d). Most shallow traps generally exist at the grain boundaries.^[^ ^20^ ^]^ The Lewis acid–base interaction between Y6 or JY16 and the perovskite precursor led to better morphology with less grain boundaries of perovskite films (Figure 2b), thereby reducing the shallow traps generated at the grain boundary. In contrast, deep traps are known to be related to defects on the perovskite surface.^[^ ^21^ ^]^ The Y6‐ and JY16‐treated PeSCs showed lower deep trap tDOS than that of control PeSC with 2.17 × 10^17^ cm^−3^ eV^−1^. Electron‐rich functional groups such as S, N, and O of Y6 and JY16 can passivate halide vacancy defects by interacting with uncoordinated Pb^2+^ located at the perovskite surface, which is consistent with the XPS results (Figure 2c). The tDOS of PeSCs with Y6 and JY16 additives decreased significantly compared with that of the control PeSCs, whereas the difference in tDOS between the PeSCs with Y6 and JY16 additives was negligible. Because Y6 and JY16 have the same backbone, it is expected that the defect passivation ability between Y6 and JY16 might be similar, which is consistent with the previous analysis (Figure 4a,b).

To confirm the cause of the enhanced charge extraction ability of the PeSC with the JY16 additive compared to the one with the Y6 additive, the mobility for Y6 and JY16 was measured through the fabrication of organic field‐effect transistor devices (FETs) (Figure 4e). The FET mobility (*µ*_FET_) in the saturation regime was extracted using Equation (4):

(4) $$\mu_{\mathrm{FET}}=\frac{2L}{C_{\mathrm{ox}}W}{\left(\frac{d\sqrt{\left|I_{D}\right|}}{dV_{G}}\right)}^{2}$$

where *L* is the channel length, *C*_ox_ the capacitance, *W* the channel width, *I*_D_ the drain current, and *V*_G_ the gate voltage. *L* and *W* are 50 and 3000 µm, respectively. *V*_G_ was scanned from 60 to −20 V with the source–drain voltage (*V*_DS_) set to 30 V. The *µ*_FET_ value of Y6 was calculated as 3.55 × 10^−3^ cm^2^ V^−1^ s^−1^. The *µ*_FET_ value of JY16 was more than ten times higher value than that of Y6, 37.9 × 10^−3^ cm^2^ V^−1^ s^−1^. This may be due to the difference in packing orientation between the linear alkyl chain of JY16 and the branched alkyl chain of Y6, resulting in the superior charge extraction ability of JY16‐treated PeSCs.^[^ ^22^ ^]^ To observe the molecular packing orientation of pristine Y6 and JY16, grazing‐incidence wide‐angle X‐ray scattering measurements were performed, and the corresponding azimuthal pole figures for the (010) *π*–*π* stacking peaks were plotted (Figure S13, Supporting Information). Note that a mixed slanted and edge‐on orientation was dominant in the JY16 film, whereas Y6 showed a preferred face‐on orientation, implying that different alkyl chains may induce significant changes in the crystalline orientation. The transient photocurrent (TPC) was measured to evaluate further insight into the enhancement of the charge extraction ability of the PeSC with the JY16 additive.^[^ ^23^ ^]^ The time constants were extracted from the measured data by fitting using single‐exponential decay model. The TPC is generated by microsecond pulses of incident light on the device and can provide information regarding charge transport within the device. The TPC measurement conducted under short‐circuit conditions showed that the charge‐transfer lifetime (*τ*_tran_) decreased from 2.31 to 1.55 and 0.751 µs for the control and PeSCs with Y6 and JY16 additives, respectively (Figure 4f and **Table** **2**). The PeSC with the JY16 additive showed the greatest improvement in charge transport and charge extraction properties, which may be related to the better mobility of JY16 compared to Y6. To further analyze the improved charge extraction ability and suppressed charge recombination of the PeSCs, semicircle Nyquist plots for the PeSCs with and without Y6‐ and JY16‐treated perovskite films were fitted (Figure S14, Supporting Information). The *τ*_trans_ and *τ*_rec_ values can be obtained directly from the *f*_min_ point of the semicircle located at the given angular frequency using *τ*_trans_ and *τ*_rec_ = 1/2*πf*_min_.^[^ ^24^ ^]^ The intensity‐modulated photovoltage spectroscopy (IMVS) and intensity‐modulated photocurrent spectroscopy (IMPS) of the Y6‐and JY16‐treated PeSCs also exhibited a faster charge‐transfer lifetime and longer charge recombination lifetime. Especially, the PeSC with JY16 showed a faster transfer lifetime of 0.558 µs and slower recombination lifetime of 5.64 µs compared to control PeSC of 0.796 and 3.56 µs. The charge collection efficiencies (*η*_CE_) calculated using the relation *η*_CE_ = 1 − (*τ*_tran_/*τ*_rec_) were 90.1% with the PeSC with JY16, which is much higher than control PeSC 77.6% (Table S2, Supporting Information). These results are consistent with those obtained from TPC, which confirmed that JY16 treatment promoted charge extraction and effectively inhibited charge recombination in perovskite films.

**Table 2:** Summary of TPC parameters for control PeSC and PeSCs with Y6 and JY16 additives

Device configuration	*τ*_tran_ [_µs_]
Control	2.31
Y6	1.55
JY16	0.751

**Figure 5:**
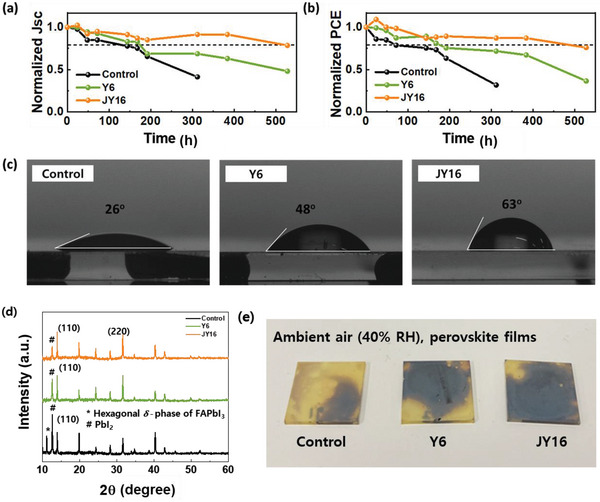
Moisture stability test of PeSCs. Evolution of normalized a) *J*_SC_ and b) PCE of control PeSC and PeSCs with Y6 and JY16 additives under 25% RH without encapsulation. c) Contact angle of water droplet from control and Y6‐ and JY16‐treated perovskite films. d) XRD spectra and e) images of control and Y6‐ and JY16‐treated perovskite films after keeping under 40% RH for 500 h.

Finally, the moisture stability of the control and PeSCs with Y6 and JY16 additives was investigated under 25% RH without encapsulation. The PeSCs with JY16 additive retained 76% of their initial PCE after 500 h, while the PeSCs with Y6 additive decreased to 42.6% of their initial PCE after 500 h and control PeSC reduced to 34.2% of their initial PCE after 300 h (**Figure** **5**a,b and Figure S15, Supporting Information). According to previous studies, prolonged exposure to humidity has detrimental effects on the perovskite films.^[^ ^25^ ^]^ The perovskite films are decomposed via defect states, such as halide vacancies and/or grain boundaries present inside the perovskite films, and decomposition is accelerated by moisture invasion from the external environment.^[^ ^26^ ^]^ Improved stability of the Y6 and JY16 PeSCs was attributed to the reduced defect states at the surface and grain boundaries of perovskite films, and resistance to moisture in the air resulting from the surface modification of the perovskite films. The control perovskite film showed a minimum water contact angle of 26°, exhibiting hydrophilic surface characteristics (Figure 5c). On the contrary, the JY16‐treated perovskite film showed the most hydrophobic surface due to the long‐linear *n*‐hexadecyl alkyl chain compared to the film with Y6, which has a branched 2‐ethylhexyl alkyl chain. Owing to the surface modification and defect passivation effects with Y6 or JY16 treatment, the PeSCs with hydrophobic JY16 additive showed better moisture stability compared to the PeSCs with Y6 additive by retarding moisture invasion from the external environment. Furthermore, to elucidate the degradation mechanism of perovskite films under moisture conditions, XRD analysis of control, Y6‐, and JY16‐treated perovskite films were performed after maintaining 40% RH over 500 h (Figure 5d). Peaks of PbI_2_ at 2*θ* = 12.6° and *δ*‐phase of FAPbI_3_ at 2*θ* = 11.2° emerged significantly in the control perovskite film, indicating that phase decomposition by moisture infiltration into the perovskite film was accelerated in the control perovskite film, which exhibited the most hydrophilic surface properties. Furthermore, when the control, Y6‐, and JY16‐treated perovskite films were stored at 40% RH, the most severe damage was identified in the control sample, consistent with the XRD analysis results (Figure 5d,e). Meanwhile, the encapsulated control, and JY16‐treated PeSC exhibited no significant degradation when kept in the N_2_ glovebox (Figure S16, Supporting Information). Both the control, and JY16‐treated PeSC maintained their initial PCEs after 1000 h. Therefore, the inclusion of JY16 into perovskite films helps prevent irreversible degradation of perovskite films under moisture conditions.

## 3 Conclusion

In conclusion, a new n‐type conjugated small‐molecule additive, JY16, with the same backbone as Y6 and long‐linear *n*‐hexadecyl alkyl side chains was synthesized to efficiently passivate the defect states of perovskite films and improve the moisture stability of PeSCs. We conducted a comparative study of the control PeSC and PeSCs with Y6 and JY16 additives to determine their effects on the devices. The PeSC with JY16 additive showed higher PCE of 21.35% without hysteresis behavior owing to excellent defect passivation capability and superior mobility of JY16 molecules. The defect passivation capability of JY16 additive in perovskite was confirmed through SEM, XPS, SCLC, ideal factor (light intensity‐dependent *V*_oc_), and frequency‐dependent capacitance measurements. To confirm the enhanced charge extraction ability of JY16 additive in perovskite, FET mobility, TPC, IMVS, and IMPS measurements were performed, and the results were compared. Furthermore, it was found that the hydrophobic nature of JY16, which has long‐linear side *n*‐hexadecyl alkyl chain, contributes to better moisture stability of the PeSCs with JY16 additive (>76% PCE retention under 25% RH without any encapsulation). We believe that the proposed organic semiconducting additive will prove to be crucial for achieving highly efficient and moisture‐stable inverted‐structured PeSCs.

## Conflict of Interest

The authors declare no conflict of interest.

## Supporting information

Supporting Information

## Data Availability

The data that support the findings of this study are available in the Supporting Information of this article.
