# Supplementary material for: Efficient and Moisture‐Stable Inverted Perovskite Solar Cells via n‐Type Small‐Molecule‐Assisted Surface Treatment
Source: Adv Sci (Weinh). 2022 Nov 23;10(3):2205127. doi: 10.1002/advs.202205127 (PMC9875621; doi:10.1002/advs.202205127)
Supplement: Supplementary file 1 — Supporting Information [file ADVS-10-2205127-s001.pdf]

Supporting Information

Efficient and Moisture-stable Inverted Perovskite Solar Cells via *n*-type Small-molecule-assisted Surface Treatment

Ji A Hong, Mingyu Jeong, Sujung Park, Ah-Young Lee, Hye Seung Kim, Seonghun Jeong, Dae Woo Kim, Shinuk Cho, Changduk Yang* and Myoung Hoon Song**

Experimental Section

Synthesis of 5,6-dinitro-4,7-bis(6-undecylthieno[3,2-b]thiophen-2-yl)benzo[c][1,2,5]thiadiazole (**1**): Compound **1** was synthesized as per literature. [1]

*Synthesis of 12,13-dihexadecyl-3,9-diundecyl-12,13-dihydro-[1,2,5]thiadiazolo[3,4-e]thieno[2'',3'':4',5']thieno[2',3':4,5]pyrrolo[3,2-g]thieno[2',3':4,5]thieno[3,2-b]indole (**2**):* To a two-neck round-bottom flask, compound **1** (4 g, 4.93 mmol) and triethyl phosphite (30 mL) were dissolved in anhydrous *o*-dichlorobenzene (30 mL) and purged with argon for 20 min. After stirring overnight at 180 °C, the reaction mixture was extracted with dichloromethane, dried over $MgSO_4$, and concentrated under reduced pressure. This orange-colored residue was dissolved in anhydrous DMAc; then KOH (1.66 g, 29.58 mmol), KI (4.09 g, 24.65 mmol), and 1-bromohexadecane (6.02 g, 19.72 mmol) were added and purged with argon for 20 min. The mixture was heated to reflux overnight and extracted with dichloromethane. The combined organic phase was washed with distilled water, dried with $MgSO_4$, and concentrated under reduced pressure. Further purification *via* silica gel column chromatography was performed using hexane/dichloromethane (3:1, v/v) to afford compound **2** as an orange solid (2.44 g, 41.3% yield). ^{1}H NMR (400 MHz, $CDCl_3$), δ (ppm): 7.01 (s, 2H), 4.62 (t, 4H), 2.82 (t, 4H), 1.87 (m, 8H), 1.26 (m, 84H), 0.86 (m, 12H).

*Synthesis of 12,13-dihexadecyl-3,9-diundecyl-12,13-dihydro-[1,2,5]thiadiazolo[3,4-e]thieno[2'',3'':4',5']thieno[2',3':4,5]pyrrolo[3,2-g]thieno[2',3':4,5]thieno[3,2-b]indole-2,10-dicarbaldehyde (**3**):* To anhydrous DCE (5 mL) in a two-neck round-bottom flask, DMF (3.5 mL) and $POCl_3$ (4.2 mL) were added at 0°C. After stirring for 1 h, the Vilsmeier reagent was

added dropwise to the solution of compound **2** (0.9 g, 1.08 mmol) in DCE (20 mL), and the reaction mixture was heated to reflux overnight. After cooling to room temperature, the mixture was poured into saturated $NaHCO_3$ solution and extracted with dichloromethane. The combined organic layers were dried with $MgSO_4$ and concentrated under reduced pressure. Further purification *via* silica gel column chromatography was performed using hexane/dichloromethane (1:1, v/v) to afford compound **3** as an orange solid (1.26 g, 92.9% yield). ^{1}H NMR (400 MHz, $CDCl_3$), δ (ppm): 10.13 (m, 2H), 4.65 (t, 4H), 3.18 (t, 4H), 1.90 (m, 8H), 1.25 (m, 84H), 0.88 (m, 12H).

*Synthesis of 2,2'-((2Z,2'Z)-((12,13-dihexadecyl-3,9-diundecyl-12,13-dihydro-[1,2,5]thiadiazolo[3,4-e]thieno[2'',3'':4',5']thieno[2',3':4,5]pyrrolo[3,2-g]thieno[2',3':4,5]thieno[3,2-b]indole-2,10-diyl)bis(methaneylylidene))bis(5,6-difluoro-3-oxo-2,3-dihydro-1H-indene-2,1-diylidene))dimalononitrile (**JY16**):* Compound **3** (150 mg, 0.12 mmol), 2-(5,6-difluoro-3-oxo-2,3-dihydro-1*H*-inden-1-ylidene)malononitrile (165.7 mg, 0.72 mmol), and pyridine (0.1 mL) were dissolved in chloroform (20 mL), and purged with argon for 20 min. After stirring overnight at 65 °C, the mixture was poured into methanol and filtered. Remaining residue was purified *via* silica gel column chromatography using chloroform as the eluent to afford **JY16** as a dark solid (169.4 mg, 84.2% yield). ^{1}H NMR (400 MHz, $CDCl_3$), δ (ppm): 8.58 (s, 2H), 8.37 (dd, 2H), 7.53 (t, 2H), 4.58 (t, 4H), 2.89 (t, 4H), 2.07 (m, 4H), 1.72 (m, 4H), 1.28 (m, 84H), 0.86 (m, 12H). ^{13}C NMR (100 MHz, $CDCl_3$), δ (ppm): 185.92, 157.39, 153.85, 152.82, 146.90, 145.09, 137.10, 134.04, 133.88, 132.57, 132.07, 130.09, 119.54, 114.70, 114.56, 114.44, 113.34, 69.24, 51.17, 32.07, 31.91, 30.91, 29.77, 27.27, 22.82, 14.27. HRMS (ESI) *m/z* 1675.79 ($C_{98}H_{118}F_4N_8O_2S_5$ calcd. for *m/z* 1675.80).

Device fabrication: The ITO substrate was prepared as in the previous report.[1] Then, 3 mmol of MeO-2PACz in ethanol was spin coated at 5000 rpm for 30 s and annealed at 100°C for 10 min under ambient conditions. 1.4 M mixed perovskite solution was fabricated by mixing the $FAPbI_3$, $MAPbBr_3$, and CsI precursor solutions in proper amount. 1.4 M $FAPbI_3$ and $MAPbBr_3$ solutions were prepared in a 4:1 (v/v) mixture of anhydrous DMF and DMSO. A CsI stock solution was prepared at a concentration of 1.5 M in DMSO. Triple-cation mixed perovskite ($Cs_{0.05}FA_{0.92}MA_{0.08}Pb(I_{0.92}Br_{0.08})_3$) was obtained by mixing the three solutions. The perovskite solution was spin coated using the dropping method at 5000 rpm for 27 s. The chloroform was then dropped as an anti-solvent 10 s before the end. Optimized Y6- and JY16-

treated PeSCs were fabricated with 0.0001 mg ml^{-1} of Y6 and JY16 in chloroform, respectively. The sample was then annealed at 100°C for 1 h. LiF (0.8 nm), C_{60} (30 nm), and Ag (80 nm) electrodes were then thermally evaporated sequentially.

Characterization: ^{1}H NMR and ^{13}C NMR spectra, SEM, AFM, XRD, HRTEM, TOF-SIMS, XPS, *J-V* curves, iIPCE, UPS, UV-vis, contact angle, the density of defect states, TPC, TPV, IMPS, IMPS were measured with the same equipment as in previous work.[2] Field-effect transistors (FETs) were fabricated on silicon dioxide (SiO_2; 200 nm, 17 nF/cm^2) covered heavily doped Si wafers with a bottom-gate, top-contract architecture. Y6 and JY16 were dissolved in chloroform at a concentration of 5 $mgml^{-1}$. The Y6 and JY16 films were deposited via spin coating at 3000 rpm for 40 s. The source and drain electrodes Al were thermally evaporated using a shadow mask. The transfer characteristics of the FETs were measured using a Keithley semiconductor parametric analyzer (Keithley 4200) in N_2 filled glovebox under dark conditions. The gate voltage V_{gs} was scanned from 60 V to -30 V with source-drain voltage V_{ds} set to 60 V.

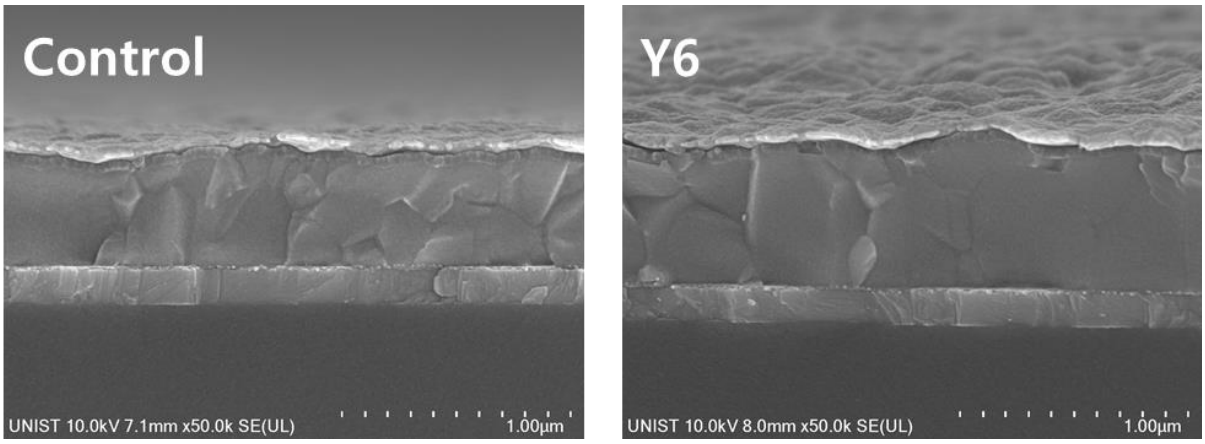

Figure S1. Cross-sectional SEM images of control PeSC and PeSC with Y6 additive.

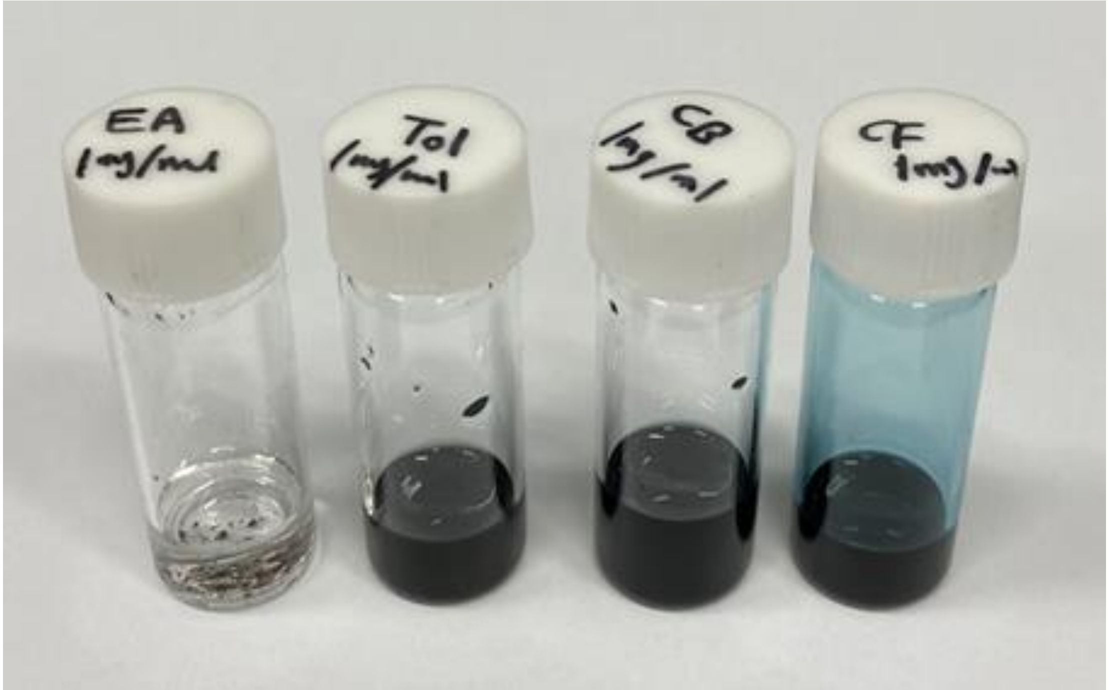

Figure S2. Solubility test of 1 mg/ml of JY16 with different anti-solvents (EA = ethyl acetate, Tol = toluene, CB = chlorobenzene, and CF = chloroform).

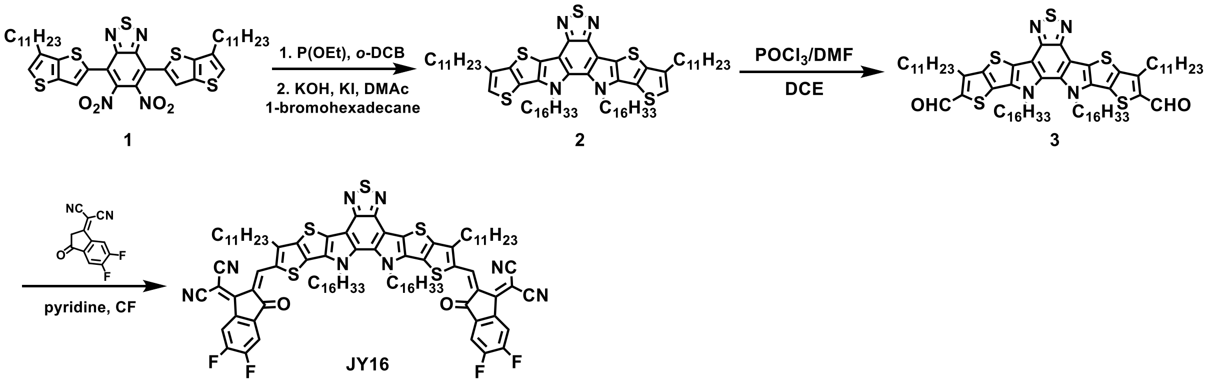

Figure S3. Synthetic procedure of JY16.

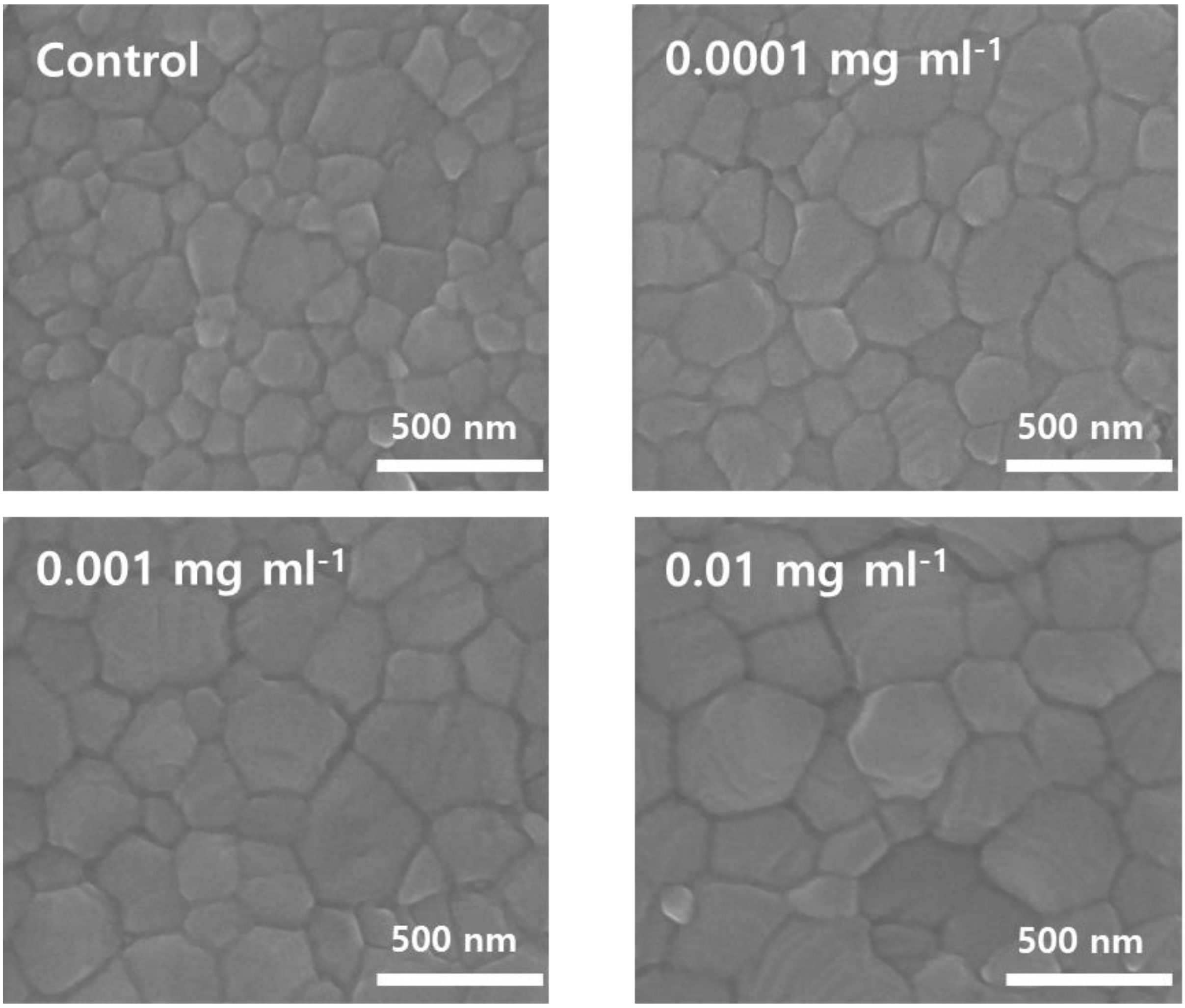

Figure S4. Top-view SEM of perovskite films with different concentrations of JY16.

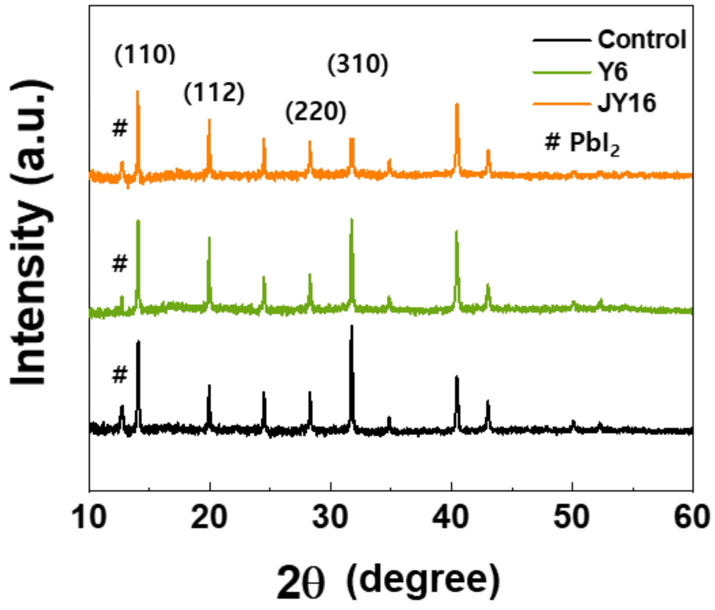

Figure S5. XRD spectra of the control and Y6- and JY16-treated perovskite films.

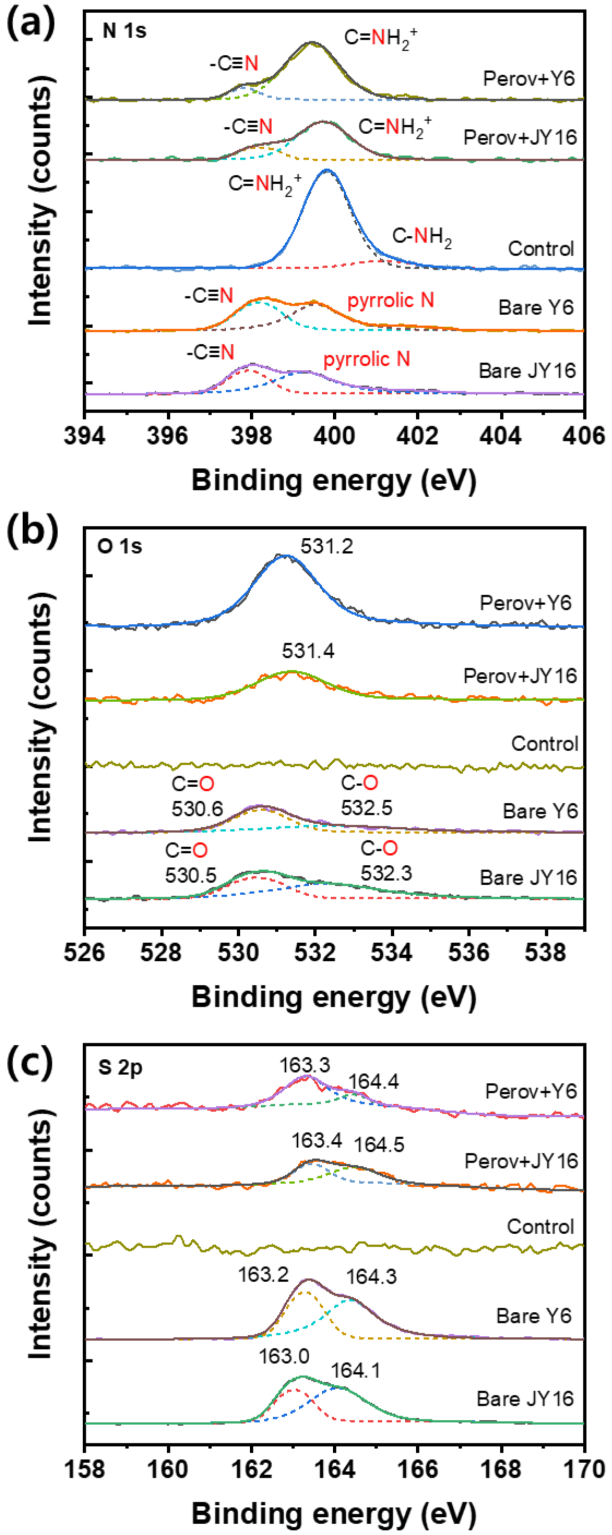

Figure S6. XPS spectra of (a) N, (b) O, and (C) S of pristine Y6, JY16 and control, Y6-, and JY16- treated perovskite films.

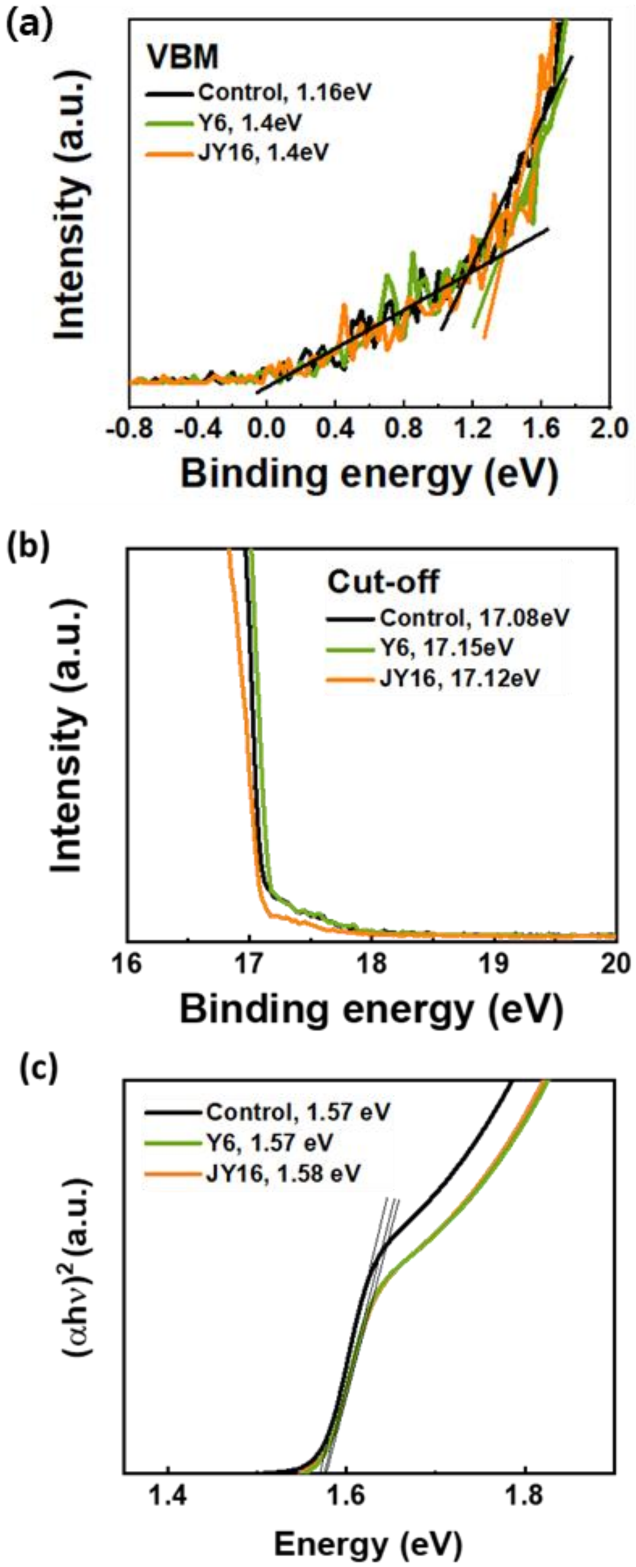

Figure S7. a) Valence band maximum energy (E_{VBM}), b) cut-off energy ($E_{cut\text{-}off}$), and c) optical bandgap of the control and Y6- and JY16-treated perovskite films.

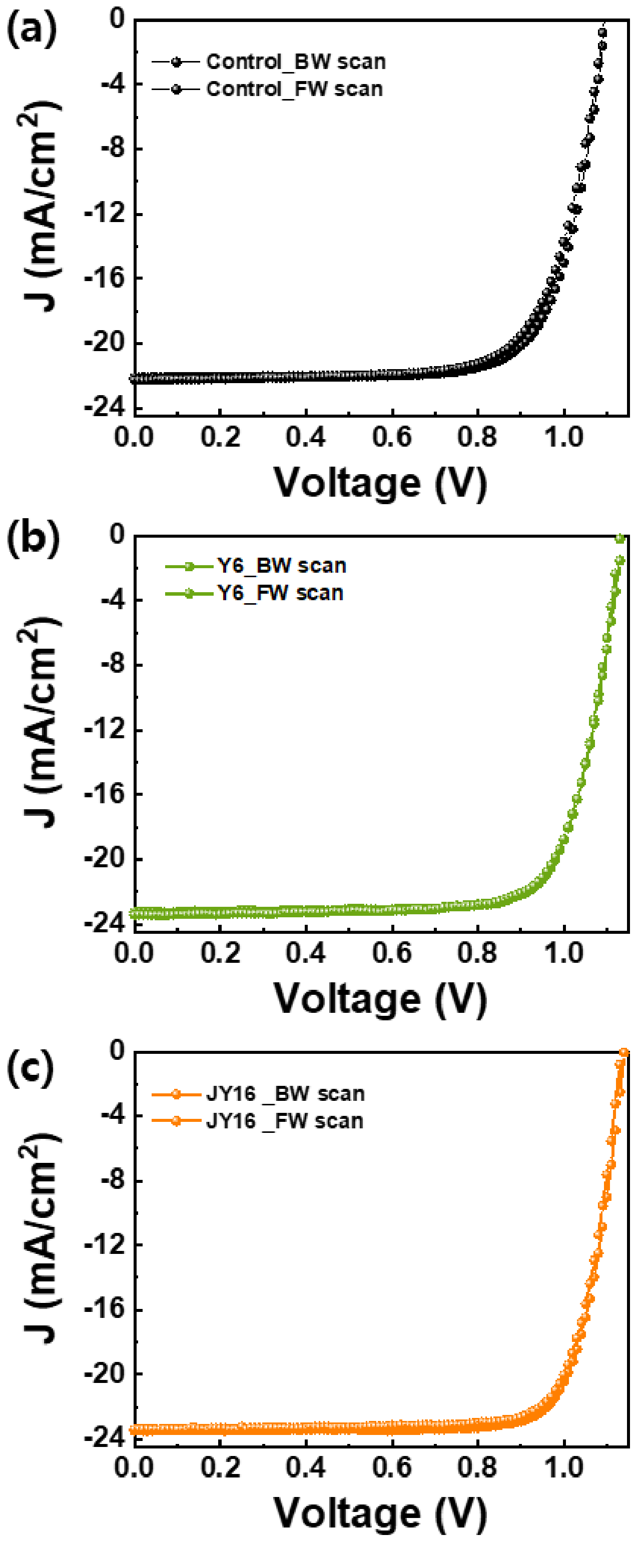

Figure S8. *J–V* hysteresis behavior of a) control PeSC and PeSCs with b) Y6 additive and c) JY16 additive.

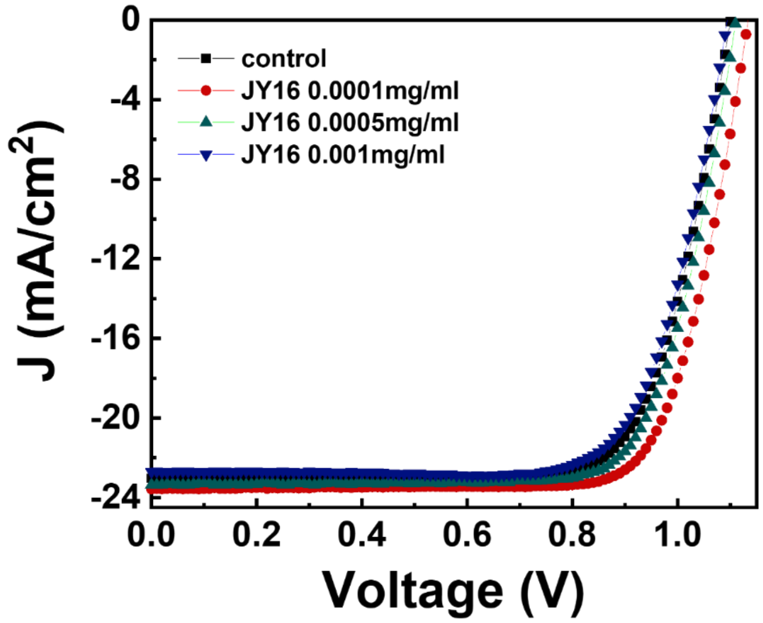

Figure S9. *J–V* characteristics of PeSCs with different concentrations of JY16.

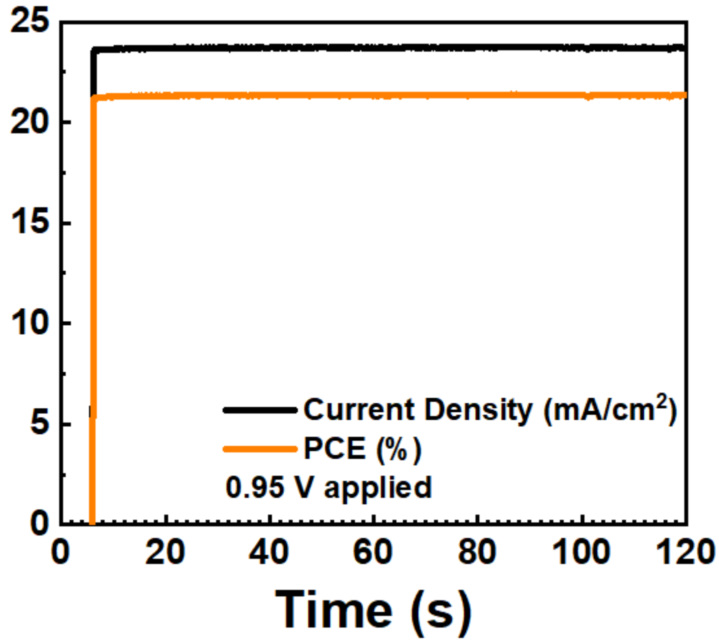

Figure S10. SPO of PeSCs with JY16 additive.

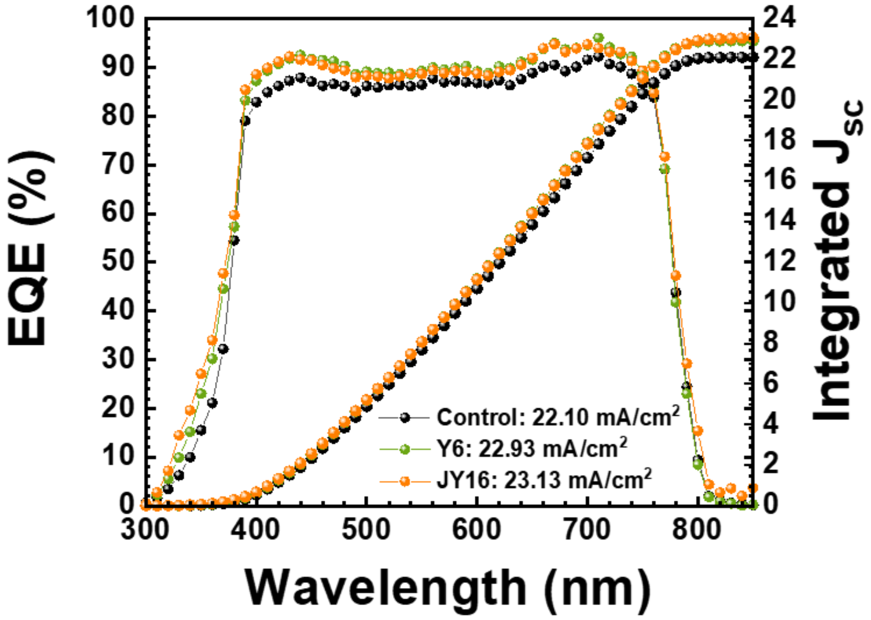

Figure S11. IPCE spectra and integrated J_{SC} of control PeSC and PeSCs with Y6 and JY16 additives.

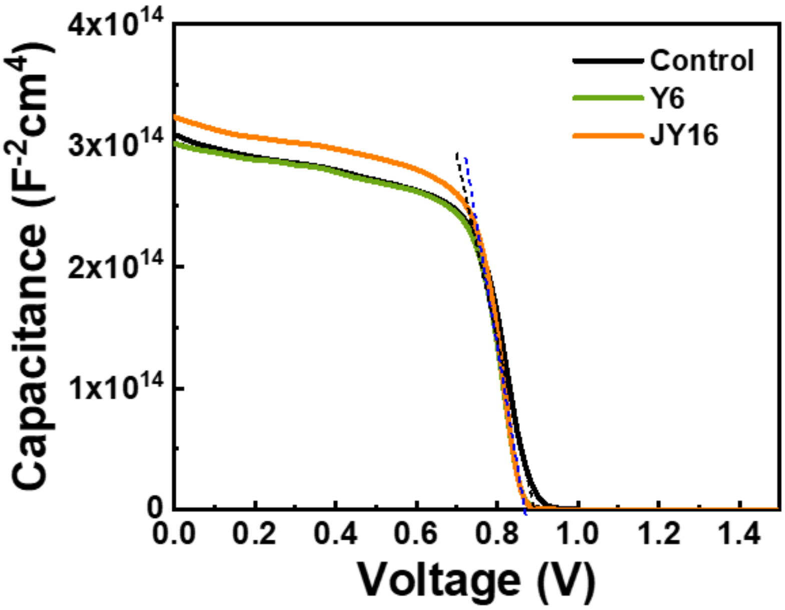

Figure S12. Mott-Schottky analysis of the control PeSC and PeSCs with Y6 and JY16 additives.

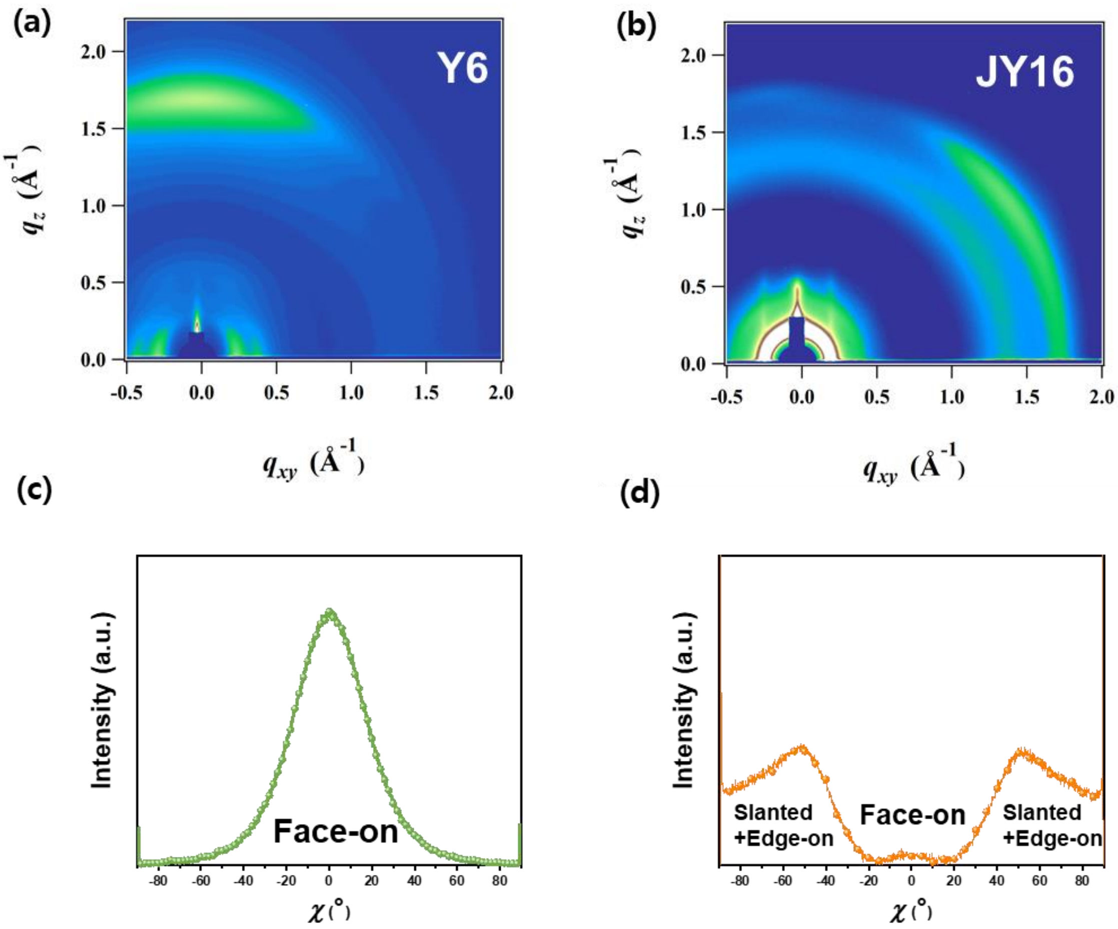

Figure S13. 2D grazing-incidence wide-angle X-ray scattering patterns of (a) Y6 and (b) JY16 and corresponding azimuthal pole figures for π–π stacking crystallites of (c) Y6 and (d) JY16.

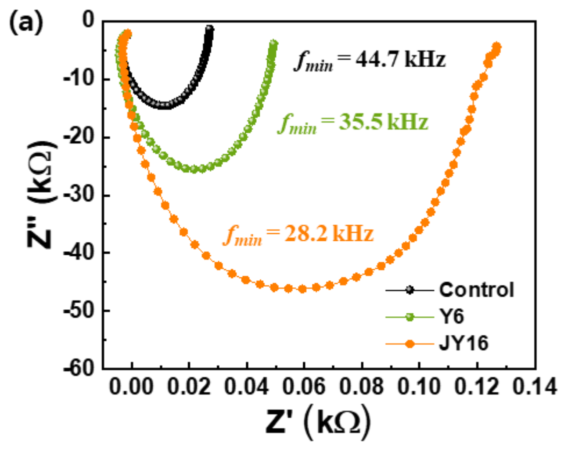

Figure S14. a) IMVS and b) IMPS spectra of control PeSC and PeSCs with Y6 and JY16 additives.

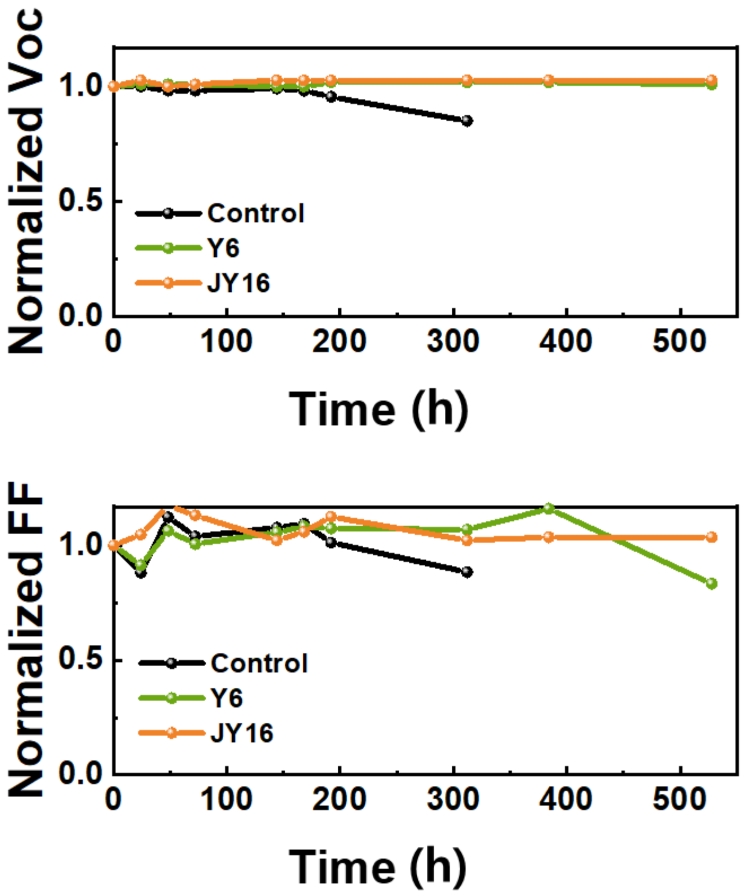

Figure S15. Evolution of normalized V_{OC} and FF of control PeSC and PeSCs with Y6 and JY16 additives under 25% RH without encapsulation.

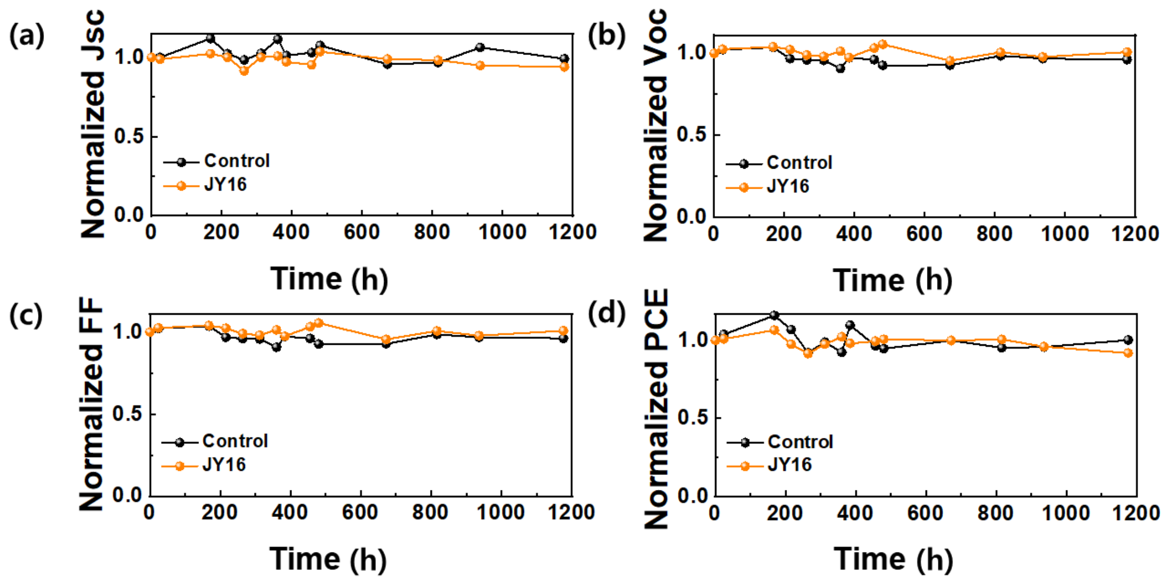

Figure S16. Evolution of normalized a) J_{SC}, b) V_{OC}, c) FF, and d) PCE of control PeSC and PeSCs with Y6 and JY16 additives kept under N_2 glovebox condition with encapsulation.

Table S1. Summary of photovoltaic parameters of PeSCs with different concentrations of JY16.

Concentration of JY16 [mg ml^{-1}]	*Jsc* [mA cm^{-2}]	*Voc* [V]	FF [%]	PCE [%]
w/o JY16	23.03	1.11	74.13	18.95
0.0001	23.56	1.14	75.93	20.39
0.0005	23.35	1.12	74.62	19.51
0.001	22.72	1.1	73.57	18.39

Table S2. Summary of IMPS and IMVS parameters for control PeSC and PeSCs with Y6 and JY16 additives.

Device configuration	τ_{tran} [μs]	τ_{rec} [μs]	η_{CE} [%]
Control	0.796	3.56	77.6
Y6	0.634	4.48	85.8
JY16	0.558	5.64	90.1

References

[1] a) G. Niu, X. Guo, L. Wang, *J. Mater. Chem. A* **2015**, *3*, 8970; b) E. Mosconi, J. M. Azpiroz, F. De Angelis, *Chem. Mater.* **2015**, *27*, 4885; c) B. Roose, K. C. Gödel, S. Pathak, A. Sadhanala, J. P. C. Baena, B. D. Wilts, H. J. Snaith, U. Wiesner, M. Grätzel, U. Steiner, A. Abate, *Adv. Energy Mater.* **2016**, *6*, 1501868; d) R. Fu, Y. Zhao, Q. Li, W. Zhou, D. Yu, Q. Zhao, *Chem. Commun.* **2017**, *53*, 1829

[2] a) D. W. Kim, E. D. Jung, C. H. Jang, J. A. Hong, H. S. Kim, Y. W. Noh, M. H. Song, *J. Power Sources* **2022**, *520*, 230783; b) A. K. Harit, E. D. Jung, J. M. Ha, J. H. Park, A. Tripathi, Y. W. Noh, M. H. Song, H. Y. Woo, *Small* **2022**, *18*, 2104933; c) E. D. Jung, A. K. Harit, D. H. Kim, C. H. Jang, J. H. Park, S. Cho, M. H. Song, H. Y. Woo, *Adv. Mater.* **2020**, *32*, 2002333; d) J. A. Hong, E. D. Jung, J. C. Yu, D. W. Kim, Y. S. Nam, I. Oh, E. Lee, J.-W. Yoo, S. Cho, M. H. Song, *ACS Appl.Mater. Interfaces* **2020**, *12*, 2417.; e) M. Jeong, I. W. Choi, K. Yim, S. Jeong, M. Kim, S. J. Choi, Y. Cho, J.-H. An, H.-B. Kim, Y. Jo, S.-H. Kang, J.-H. Bae, C.-W. Lee, D. S. Kim, C. Yang, *Nat. Photonics* **2022**, 16, 119.
